# Culture filtrate proteins from BCG act as adjuvants for cytotoxic T lymphocyte induction

**DOI:** 10.3389/fimmu.2023.1271228

**Published:** 2023-10-18

**Authors:** Satoru Mizuno, Yasushi Chuma, Yukihiro Shibuya, Shigeo Horibata, Tomoe Baba, Emi Yokokawa, Kazuhiro Matsuo

**Affiliations:** ^1^ Research and Development Department, Japan BCG Laboratory, Kiyose, Tokyo, Japan; ^2^ Institute for Vaccine Research and Development, Hokkaido University, Sapporo, Hokkaido, Japan

**Keywords:** cytotoxic T lymphocytes, culture filtrate protein, tuberculosis, Mycobacterium bovis bacilli Calmette-Guerin, immunotherapy

## Abstract

*Mycobacterium bovis* bacilli Calmette-Guerin (BCG) is a licensed vaccine against tuberculosis. It requires attenuated live bacteria to be effective, possibly because actively secreted proteins play a critical role in inducing anti-tuberculosis immunity. BCG also functions as an effective adjuvant. Moreover, the effects of BCG components as adjuvants are not important as those of attenuated live BCG, which is used in cancer immunotherapy. However, the BCG secreted proteins have not been paid attention in anticancer immunity. To understand mycobacterial secreted proteins’ function, we investigate immune responses to BCG culture filtrate proteins (CFP). Here, CFP strongly induce both antigen-specific CD4+ T cells and specific CD8+ T cells, which may be functional cytotoxic T lymphocytes (CTLs). In this study, we clearly demonstrate that CFP acts as an adjuvant for CTL induction against specific co-administered proteins and propose CFP as a new protein adjuvant. The CTL response shows potent anticancer effects in mice. These findings could provide insight into the contribution of mycobacterial secreted proteins in both anticancer and antimycobacterial immunity.

## Introduction

*Mycobacterium bovis* bacilli Calmette-Guerin (BCG) is the only licensed tuberculosis (TB) vaccine. Although this bacterium has been used as an effective vaccine against infant TB for a long time, its efficacy in adult pulmonary TB has been limited. This has rendered it ineffective in the global fight against TB (1, 2).

Heat-killed BCG exhibited no protective effect against *Mycobacterium tuberculosis* (Mtb) infection in mice (3). Thus, attenuated live bacteria are required to ensure BCG vaccine efficacy, possibly because secreted protein antigens produced by attenuated live BCG play an important role in inducing protective immunity against TB. Secreted proteins have been purified from the culture filtrate of BCG (4–6) or Mtb (7) and their genes cloned and expressed to characterize their immunogenicity (8–12). Furthermore, specific secreted protein preparations from Mtb and BCG have exhibited protective effects in mice (13) and guinea pigs (14). Major secreted Mtb proteins Ag85A and Ag85B were putative protective antigens, and the *Ag85A* gene was inserted in the modified vaccinia Ankara strain to produce the MVA85A vaccine (15). This candidate vaccine was clinically tested (16) but was not efficacious in BCG-vaccinated newborn babies boosted with the MVA85A vaccine. However, *Ag85B* gene was introduced into BCG to construct a recombinant BCG vaccine (rBCG30), which was evaluated for efficacy in guinea pigs (17). This recombinant BCG vaccine showed higher protection against Mtb infection than the parental BCG vaccine. In addition, several secreted proteins were examined as candidate protective antigens. However, individual proteins did not show comparable protective activity to the BCG vaccine. Consequently, the hypothesis that the secreted proteins are critical antigens for Mtb protection has not been proven yet.

Mycobacterial cell components also function as adjuvants. Freund’s complete adjuvant contains dead Mtb cells. It has been widely used against protein antigens and as a cytotoxic T lymphocyte (CTL) inducer against various peptide antigens in animal experiments. Therefore, BCG cell components (18–21), lipid components (22), and glycolipids (22, 23) were extracted from the BCG cell wall and assessed as adjuvants for use in anticancer immunotherapy. However, these have not been put into practical use. In contrast, attenuated live BCG has been used for intravesical and intratumoral injection therapies against bladder cancer in many countries (24) and melanoma lesions in the United States (25), respectively. Thus, the full adjuvant activity of BCG must also include attenuated live bacteria when utilized in cancer immunotherapy. It is critical to fully consider the immune response to each actively produced component to better understand the adjuvant activity of the attenuated live BCG.

The Japan BCG Laboratory is a unique BCG vaccine manufacturer in Japan. The BCG culture filtrate is discarded during the vaccine production process. Here, we utilized the culture filtrate to prepare a concentrated protein fraction, namely culture filtrate proteins (CFP), and evaluated various immune responses. Moreover, we analyzed the CFP function in immune induction and clearly demonstrated that CFP acts as a form of adjuvant for the induction of CTL against co-administrated proteins. Therefore, we hypothesized that CFP is a new protein adjuvant component produced by mycobacteria.

## Methods

### Bacterial strain and culture medium

The *M. bovis* BCG Tokyo 172 strain was cultured on Sauton medium (26) as a pellicle according to the approved production procedure.

### Preparation of CFP

A lot of BCG culture filtrate (80 L), which was a by-product from the validated BCG vaccine production process, was collected at the early stationary phase using centrifugal ultrafiltration (Pelicon cassette; 5 kDa; Merck Millipore Co. Ltd., Burlington, MA, USA). Saturated ammonium sulfate solution was added to obtain a 50% saturation. After storage at 9°C for 16 h, precipitated proteins were recovered via centrifugation at 10,440 × *g* and 4°C for 30 min. The residue was washed three times with the 50% saturated ammonium sulfate solution and dissolved in phosphate-buffered saline (PBS). After desalting through dialysis, the protein amount was determined using a Pierce BCA Protein Assay Kit (Thermo Fisher Scientific, Waltham, MA, USA) to obtain 1.04 g CFP. These procedures are repeated for preparation of another lot of CFP. Sodium dodecyl sulfate-polyacrylamide gel electrophoresis (SDS-PAGE) and two-dimensional PAGE were performed as previously described (27). A flask of BCG culture on the Sauton medium was heated at 90°C for 1 hour. After heat treatment, residual BCG was filtered out and then proteins were purified as described for CFP. This preparation of CFP with heat-treatment was named CFPH.

### Animal experiments

Totally 277 C57BL/6j and 30 BALB/c female six-week-old mice were purchased from Japan SLC, Inc., Shizuoka, Japan. OT-I mice were provided by the National Institute of Infectious Diseases, Japan and obtained through home breeding in the animal experimentation facility of Japan BCG Laboratory. Animals were kept under specific pathogen-free conditions. All animal experiments were approved by the Institutional Animal Care and Use Committee of Japan BCG Laboratory.

### IFN-γ ELISPOT assay for PepA-specific cellular immune responses

C57BL/6j mice were subcutaneously inoculated with 1 × 10^6^ colony forming units of BCG in 0.1 mL PBS. Six weeks after BCG priming, the mice were boosted with 50 µg CFP, PPD, or CFPH three times at two-week intervals. The mice were euthanized two weeks after the last boost. Next, the lungs were removed and used to prepare cell suspensions at 5 × 10^6^ cells/mL in the culture medium. Lung cells were suspended to 2 × 10^5^ cells/well in RPMI-1640 medium (Fujifilm Wako, Tokyo, Japan) with 10% fetal bovine serum (FBS) (Thermo Fisher Scientific), 1% penicillin/streptomycin (Thermo Fisher Scientific), and 50 µM 2-mercaptoethanol (2ME) (Thermo Fisher Scientific). They were subsequently stimulated with 10 µg/mL GM10 peptide [CD8+ T cell epitope of mycobacterial Mtb32a protein (PepA), GAPINSATAM (28)], or 10 µg/mL peptide25 [CD4+ T cell epitope of Ag85B, FQDAYNAAGGHNAVF (29)] on ELISPOT plates (Mabtech, Nacka Strand, Sweden). The cells were incubated at 37°C for 24 h. Next, plates were washed with PBS and incubated with 1 µg/mL biotinylated anti-mouse IFN-γ mAb R4-6A2 (Mabtech) in PBS containing 0.5% FBS for 2 h at 25°C. Then, 100 µL streptavidin-alkaline phosphatase (Mabtech) was added to each well at a 1/500 dilution. After 1 h, spots were visualized using a peroxidase stain diaminobenzidine kit (Nacalai Tesque, Kyoto, Japan) and counted using the KS ELISPOT reader (ZEISS, Oberkochen, Germany).

### Antigens and reagents

OVA was purchased from Merck Millipore. MHC class I-binding OVA-peptide (SIINFEKL) and B16 melanoma peptide TRP-2 (VYDFFVWL) were purchased from Medical & Biological Laboratories Co., Ltd. (Tokyo, Japan). Monoclonal antibodies, including anti-CD11c (N418), anti-MHC class I (AF6.88.5.5.3), anti-MHC class II (M5/114.15.2), and CD86 (B7-2), were purchased from Thermo Fisher Scientific to analyze BMDC surface antigens.

### Preparation of cells

EL-4 (Public Health England, Salisbury, UK) and Colon-26 (Tohoku University Cell Resource Center for Biomedical Research/Cell Bank, Miyagi, Japan) cells were grown in RPMI-1640 medium with 10% FBS, 1% penicillin/streptomycin, and 50 µM 2ME. EG7 cells (ATCC CRL-2113), which are the OVA gene transfectants of EL4 cells, were grown in RPMI-1640 medium with 40 µg/mL G-418 sulfate (Fujifilm Wako). Furthermore, B16F10 cells (ATCC CRL-6475) were grown in D-MEM (Fujifilm Wako) with 10% FBS, 1% penicillin/streptomycin, and 2ME. BMDCs were prepared from C57BL/6j mice as previously described (30). Brifely, the mouse bone marrow cells were grown in an RPMI-1640 medium supplemented with 10 ng/mL recombinant mouse granulocyte/macrophage colony-stimulating factor (rGM-CSF) (Thermo Fisher Scientific) and recombinant mouse IL-4 (rmIL-4) (R&D Systems, Minneapolis, MN, USA) at 37°C under 5% CO_2_ in a humidified incubator. On Day 3, nonadherent cells were collected from the supernatant, gently washed, and added to fresh RPMI-1640 medium supplemented with 10 ng/mL rGM-CSF and 4 ng/mL rmIL-4. On Day 6, nonadherent cells were collected via centrifugation, resuspended in fresh RPMI-1640 medium with rGM-CSF and rmIL-4, and cultured for an additional 24 h in cell culture dishes. CD11c expression was confirmed in the cells, and CD11c positivity was >92%.

### *In vitro* analysis of OVA-peptide presentation in BMDCs

Immature BMDCs (1 × 10^6^ cells/mL) were plated in 96-well round-bottom tissue culture plates with 10 µg/mL OVA mixed with CFP (200 µg/mL) or without. The cells were subsequently cultured at 37°C under 5% CO_2_ for 24 h. OVA-treated BMDCs were cultured with 10 µg/mL mitomycin C (MMC) (Merck Millipore) for 45 min. The cells were washed, suspended in fresh medium with 2.5 × 10^6^ cells/mL CD8+ T cells from OT-I mice, and cultured for 24 h under the same conditions. CD8+ T cells were isolated from the spleens of OT-I mice using an EasySep Mouse CD8+ T cell Isolation Kit (Veritas, Tokyo, Japan). Purities of the CD8+ T cells were >95%, as determined using flow cytometry (FACSCalibur, BD Bioscience, San Jose, CA, USA). The concentration of IL-2 in the culture supernatants was measured according to the manufacturer’s instructions using an enzyme-linked immunosorbent assay (ELISA) kit (R&D Systems).

### Immunization

On Days 0 and 10, C57BL/6j mice were immunized through subcutaneous injection of OVA (10 µg/mouse) or B16F10 cell lysate (equivalent to 1 × 10^6^ cells/mouse) mixed with or without CFP (200 µg/mouse) on the abdomen. BALB/c mice were immunized with Colon-26 cell lysate (equivalent to 1 × 10^6^ cells/mouse) using the same procedure as the OVA and B16F10 lysate. To prepare a tumor cell lysate, B16F10 or Colon-26 cells were divided into 1.5-mL tubes (5 × 10^7^ cells per 500 µL PBS/tube) and disrupted using a Bioruptor (Cosmo Bio, Tokyo, Japan). Disrupted cells were centrifuged at 14,000 × *g* for 15 min at 4°C, and the supernatant was used as a cell lysate (100 µL is equivalent to 1 × 10^6^ cells).

### ELISA for antigen-specific IFN-γ production

Immune mouse spleens were removed seven days after the second immunization with OVA. Next, spleen cells (1 × 10^7^) were cultured *in vitro* with OVA-peptide (10 µg/mL) at 37°C for two days. IFN-γ production of the spleen cells in the culture supernatant was quantified using an ELISA kit (R&D Systems) according to the manufacturer’s instructions.

### CTL assay

Seven days after the second immunization with OVA or B16F10 cell lysate, immune mouse spleen cells (1 × 10^7^) were cultured *in vitro* with MMC-treated EG7 cells (6 × 10^5^) or TRP-2 peptide (10 µg/mL), respectively. The MMC-treated EG7 cells were obtained by culturing EG7 cells in 50 µg/mL MMC for 45 min and washing them. Floating cells were collected after five days of culture, and lymphocytes were prepared using Lympholyte M Cell Separation Media (TCP Analytical group, Isle of Palms, SC, USA) for effector cells. EG7 and B16F10 or TRP2 peptide-pulsed EL-4 cells were used as target cells for OVA- and TRP-2-specific cytotoxicity, respectively. The TRP-2 peptide-pulsed EL-4 cells were obtained by culturing EL-4 cells in 5 µg/mL TRP-2 for 90 min and washing them. The antigen-specific cytotoxicity evaluation was performed using a 7-AAD/CFSE Cell-Mediated Cytotoxicity Assay Kit (Cayman Chemical Company, Ann Arbor, MI, USA). Target cells were stained with 5- (6)-carboxyfluorescein diacetate succinimidyl ester (CFSE) and incubated at 37°C for 30 min. CFSE-stained target cells were incubated with various effector cells to target cell ratios at 37°C for 3 h. They were subsequently stained with 7-amino-actinomycin D (7AAD). Finally, CFSE+7AAD+ cells were measured using FACSCalibur (BD Bioscience). Data were analyzed using the FlowJo software (BD Biosience).

### Tumor cell growth suppression assay

The prevention model was constructed by subcutaneously administering EG7 (1 × 10^6^ cells/mouse: n = 10), B16F10 (1 × 10^5^ cells/mouse: n = 20) and Colon-26 cells (2.5 × 10^5^ cells/mouse: n = 10) in OVA-, B16F10 cell lysate-, and Colon-26 cell lysate-immunized mice, respectively. The cells were administered on the ventral side seven days after the second immunization. Tumor size was measured twice weekly using a microcaliper and calculated as follows: tumor size (mm^3^) = length × width^2^. To ensure a humane endpoint, the animal was considered dead and euthanized by hyperanesthesia if body weight decreased by > 10% in one week.

To construct the treatment model, EG7 (1 × 10^6^ cells/mouse: n = 10) or B16F10 (1 × 10^5^ cells/mouse: n = 10) were subcutaneously transplanted on the ventral side of C57BL/6j mice. On Days 7, 10, 14, 17, 21, and 24 after tumor transplantation, OVA (10 µg/mouse) or B16F10 cell lysate (equivalent to 1 × 10^6^ cells/mouse) mixed with or without CFP (200 µg/mouse) were intradermally injected around the tumor for the EG7- or B16F10-challenged mice, respectively. Assessment of the tumor size and humane endpoint were as described. For the improved treatment model, BCG Tokyo (0.1 mg/mouse: n = 6) (Japan BCG Laboratory, Tokyo, Japan) was subcutaneously administered on the back of the neck in C57BL/6j mice. Six weeks after BCG injection, EG7 cells (1 × 10^6^ cells/mouse) were subcutaneously transplanted into the left ventral side on Day 0. On Days 7, 10, 14, 17, 21, and 24 after EG7 cell transplantation, OVA (10 µg/mouse) mixed with or without CFP (200 µg/mouse) was intradermally injected around the tumor to treat the mice. Assessment of the tumor size and humane endpoint were as described.

### Statistical analysis

Data are presented as means ± SD. Statistical analyses were performed through the Mann-Whitney *U* test using GraphPad Prism 9 (GraphPad, Software La Jolla, CA, USA). Significant differences compared with the control are indicated by asterisks. P < 0.05 was considered statistically significant. *P < 0.05; **P < 0.01; n.s., not significant.

## Results

### Antigen-specific CD8+ T cell induction using CFP as adjuvant

A flow chart describing the preparation of the BCG CFP is shown in **Supplementary Figure 1**. In this study, two-dimensional gel electrophoresis revealed >100 protein spots (**Supplementary Figure 1**). CFP induced PepA (GM10 CTL epitope)-specific CD8+ T cells in mice primed with the BCG Tokyo vaccine strain and intranasally boosted with CFP three times. Although purified protein derivatives (PPD)-specific CD4+ T cell induction was observed (**Supplementary Figure 2**), this result was not observed with PPD boosted using gamma-interferon (IFN-γ) enzyme-linked immunospot (ELISPOT) assays. Granzyme B expression indicated that the induced CD8+ T cells would be functional CTLs. PPD and CFP possess similar components. Therefore, we suspected that the difference could be due to heat treatment during PPD preparation. To clarify this point, we tested the effect of CFP heat treatment (**Supplementary Figure 1**) in the same immunization protocol. CFPH did not induce the PepA-specific CTLs (**Supplementary Figure 2**), suggesting that the presence of a higher-order protein structure is involved in CFP activity. Based on these observations, we hypothesized that the PepA-specific CTLs might be induced by an unknown protein.

**Figure 1:**
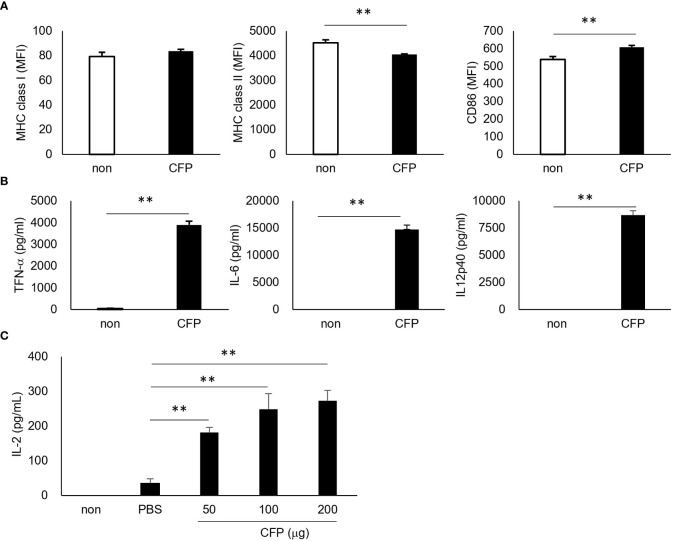
Activation of BMDCs and increased antigen presentation to CD8+ T cells. **(A)** Expression of the surface markers of OVA-BMDC, as assessed via fluorescence-activated cell sorting (FACS). **(B)** Concentration of cytokines in the cultured supernatant, as assessed via ELISA. **(C)** The concentration of IL-2 in the supernatant of coculture of OVA/CFP-incorporated BMDCs and CD8+ T cells from OT-I mice, as measured via ELISA. Representative results of three independent experiments are shown. ** P < 0.01, as determine through the Mann-Whitney *U* test.

To understand CD8+ T cell activation by CFP, we first examined its action *in vitro* by culturing bone marrow-derived dendritic cells (BMDCs) from C57BL/6j mice with CFP. The expression of major histocompatibility complex (MHC) class I was not significantly different, whereas that of MHC class II was significantly reduced compared with the BMDCs cultured without CFP. In contrast, CFP activated CD86, a ligand of CTLA4 on T cells (31) (**Figure 1A**). In addition, BMDCs showed significant production of proinflammatory cytokines such as tumor necrosis factor (TNF)-α, interleukin (IL)-6, and IL-12p40 (**Figure 1B**). Moreover, an inhibitory cytokine IL-10 was produced (data not shown). The lower expression of MHC class II on BMDCs may be due to the increased IL-10 production by these cells. Furthermore, ovalbumin (OVA)-specific CD8+ T cell activation was examined by coculture with BMDC incorporated with CFP and OVA, which mimicked an *in vivo* CTL reaction. In this system, CFP clearly enhanced IL-2 production in the BMDC with a dose-dependency (**Figure 1C**), suggesting that CFP-induced CD8+ T cell activation may be involved in the activation of antigen presentation cells (APCs) such as dendritic cells.

**Figure 2:**
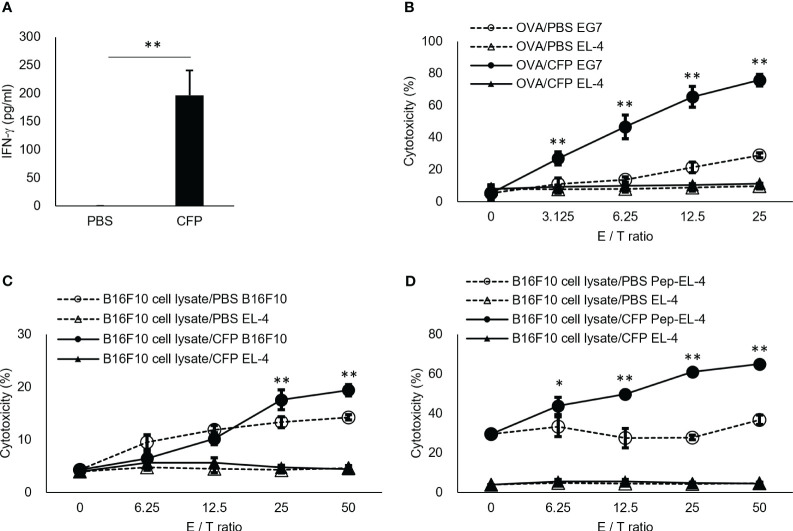
Antigen-specific CD8+ T cell induction by CFP. **(A)** Spleen cells were cultured *in vitro* with OVA-peptide and IFN-γ in the culture supernatant was assessed via ELISA. **(B, C)** Splenocytes were cultured with MMC-treated EG7 cells **(B)** or TRP-2 **(C)**. Purified lymphocytes were used for effector cells. EG7 **(B)** and B16F10 **(C)** or TRP2 peptide-pulsed EL4 **(D)** cells were used as target cells, and EL-4 cells as the target cell control. Antigen-specific cytotoxicity evaluation was performed via 7-AAD assay. CFSE+7AAD+ cells were measured using FACS. Dotted lines with open triangles and circles indicate results of coculture of EL-4 cells and EG7 **(B)**, B16F10 **(C)**, or TRP2 peptide-pulsed EL4 **(D)** cells with effector cells from control mice, respectively. Solid triangles and circles indicate results of coculture of EL-4 cells and EG7 **(B)**, B16F10 **(C)**, or TRP2 peptide-pulsed EL4 **(D)** cells with effector cells from the antigen/CFP-immunized mice, respectively. The values represent the mean and standard deviation of the triplicate cultures. Representative results of three independent experiments are shown. * P < 0.05, ** P < 0.01, as determined through the Mann-Whitney *U* test.

Here, we also examined CFP action *in vivo*. To directly prove our hypothesis, we mixed OVA, which is a protein antigen unrelated to BCG, with CFP to examine OVA-specific CTL induction. Although EL4 cells were not lysed as a target cell control, EG7 cells were killed in an effector vs target ratio-dependent manner (**Figure 2A**), indicating that the OVA-specific CTLs were highly induced. IFN-γ+ T cell induction and EG7 tumor growth suppression were reproducible even when different lots of CFP were used in the independent experiments (**Supplementary Figure 3**). In addition, splenocytes from OVA/CFP-immunized mice showed enhanced IFN-γ release into the culture supernatant (**Figure 2B**). These data supported our hypothesis and clearly demonstrated that CFP acts as a CTL adjuvant. However, CFP did not show an adjuvant effect in the induction of OVA-specific antibody production (data not shown). To clarify the CTL adjuvant activity of CFP, we induced CTLs against another antigen. B16F10 melanoma cell lysate was used with CFP in the immunization of mice, and the CTL response to tyrosinase-related protein-2 (TRP-2) tumor antigen (32) was analyzed. CTL induction was demonstrated in killing assays using both B16F10 (**Figure 2C**) and peptide-pulsed EL4 cells as target cells (**Figure 2D**). This suggests that CFP can induce CTLs against various protein antigens.

### Tumor growth suppression by CFP adjuvant in prevention model

**Figure 3:**
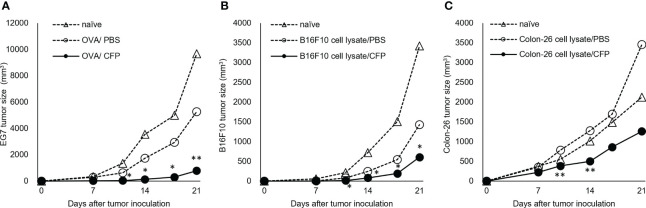
Tumor cell growth suppression by CFP in prevention models. Tumor size after injection of OVA **(A)**, B16F10 cell lysate **(B)**, or Colon-26 cell lysate **(C)** with or without CFP on Days 0 and 10. Dotted lines with open triangles and circles indicate naïve and control groups, respectively; solid circles indicate antigen/CFP-treated group. Representative results of two independent experiments are shown. *P < 0.05, ** P < 0.01, as determined through the Mann-Whitney *U* test.

To examine whether the CTL response enhanced with CFP exhibited a protective effect, we analyzed the preventive effect of CFP against tumor cell growth in mice and EG7 tumor growth was markedly suppressed by immunization (**Figure 3A**). We adopted B16F10 cells to confirm the applicability of the CFP adjuvant not for an artificial EG7 tumor but for natural cancer cell growth suppression. CFP mixed with B16F10 cell lysate was administered to mice, and the mice were subsequently challenged with B16F10 cells. The immunized mice considerably suppressed B16F10 cell growth (**Figure 3B**). Moreover, the tumor cell lysate/CFP vaccine regimen effectively suppressed the growth of the Colon-26 murine colon carcinoma cell line. BALB/c mice immunized using Colon-26 cell lysate with CFP showed reduced tumor cell growth (**Figure 3C**). Thus, the CTL adjuvant activity of CFP was unaffected by MHC class I restriction because tumor growth suppression was demonstrated in both C57BL/6 and BALB/c mice. These data suggest that antigen-specific CTL induction by the CFP adjuvant could effectively prevent the growth of various tumor cells.

### Tumor growth suppression by the CFP adjuvant in treatment models

**Figure 4:**
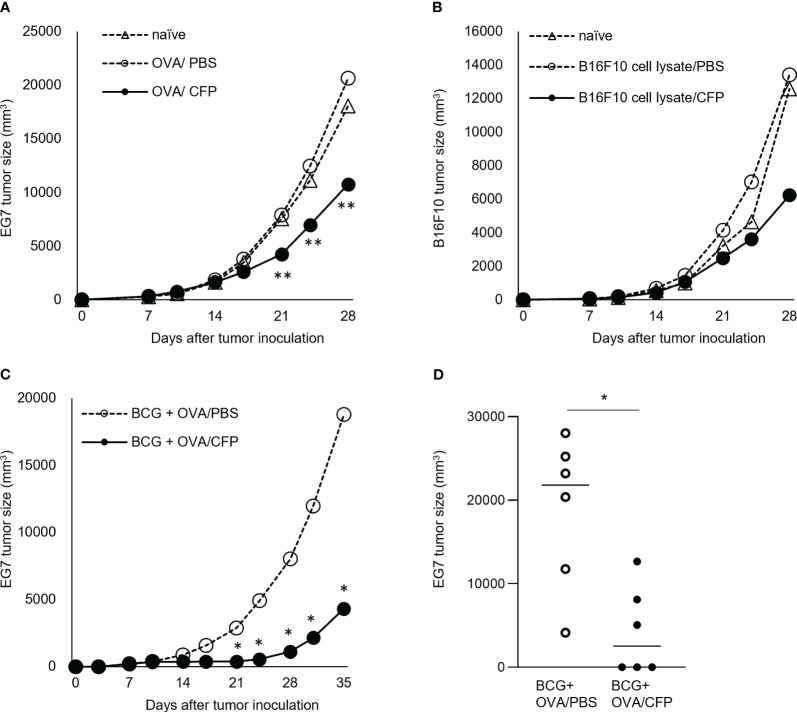
Tumor cell growth suppression by CFP in treatment models. Tumor size after transplantation of EG7 **(A)** or B16F10 **(B)** on Day 0. Tumor size after injection of OVA and CFP around the tumor site on Days 7, 10, 14, 17, 21, and 24 **(C, D)**. Dotted lines with open triangles and circles indicate the naïve mice and control groups, respectively; solid circles indicate the antigen/CFP group. **(D)** Tumor size on Day 35 is indicated. Representative results of three independent experiments are shown. *P < 0.05, ** P < 0.01, as determined through the Mann-Whitney *U* test.

Mice were inoculated with EG7 cells and then treated with the OVA/CFP mixture several times from Day 7 post inoculation. The EG7 tumor size was considerably reduced in mice treated with the OVA/CFP mixture from 11 days post inoculation (**Figure 4A**). In addition, this treatment effect was enhanced by BCG priming (**Figure 4C**). Three out of six mice treated with the OVA/CFP mixture showed tumor regression 24 days after EG7 cell transplantation (**Figure 4D**). We applied the regimen in the B16F10 treatment models similar to the EG7 experiment to elucidate the tumor suppressive effect of CTLs. The B16F10 cell lysate and CFP mixture-immunized mice showed suppressed melanoma cell growth (**Figure 4B**). These data indicate that CFP could have potential as a CTL adjuvant for cancer immunotherapy.

## Main findings and discussion

Here, we discovered that secreted proteins from BCG function as a CTL adjuvant. Although studies have reported that antigen-specific CTLs were induced against mycobacterial secreted proteins (33–35), the mechanism underlying this process was not elucidated. The protein could not effectively induce CD8+ T cells without an adjuvant because the protein antigen does not enter the classical MHC class I pathway of antigen presentation (36). However, CFP, which only contains proteins, successfully functioned as an adjuvant to induce CD8+ CTLs specific for the antigens, including OVA, PepA, and TRP-2. These findings suggest that this new adjuvant could contribute to CTL induction against various protein antigens.

The CTL response by CFP adjuvant against OVA or tumor cell lysate antigens shows potent anticancer effects in mice. In the cancer immunotherapy, there are drastic advances in the use of immune checkpoint inhibitors (37, 38). Because the CFP could work at T cell activation phase, combination with these drugs is attractive regimen expecting synergy of T cell activation and prevention of T cell exhaustion. Such issues are remained to be addressed.

The positive effect of the prior BCG vaccination in the EG7 tumor treatment model demonstrates that BCG priming sensitized CD4+ T cells specific for various proteins. Tuberculin proteins induce strong type 1 helper T cell responses that cause a delayed-type hypersensitivity reaction. At the CFP boost step, such memory CD4+ T cells would promptly make recall responses and may contribute to maturation of the OVA-specific CD8+ T cells. This mechanism could work with CD8+ T cell induction to generate functional CTLs.

A detailed mechanism analysis is required to better understand the CTL adjuvanticity of the BCG CFP. However, CFP is a mixture of >100 proteins. Therefore, the verification of CTL adjuvanticity for each protein and identification of the “key protein” warrant further study. To the best of our knowledge, no other study has investigated the CTL adjuvanticity of mycobacterial proteins. Several lipoproteins, which are components of CFP, were reported to act as TLR2 agonists and regulate innate immunity and APC function (39–41). Furthermore, such TLR2 engagement on CD4+ T cells increases Mtb antigen specific responses (42). However, these reports mentioned nothing about the CTL adjuvanticity of the lipoproteins. If the “key protein” is purified and identified, it could be developed as a novel CTL adjuvant for application in the production of vaccines against several infectious diseases and cancers where antigen-specific CTL function is involved. Purification and identification of major secreted proteins in CFP are underway using *in vitro* assay in which OVA-specific CD8+ T cell activation is examined by coculture with BMDC incorporated with OVA and each purified protein and candidate proteins are going to be characterized. Furthermore, this may provide insight into the contribution of the mycobacterial secreted proteins in antimycobacterial immunity.

## Data availability statement

The original contributions presented in the study are included in the article/**Supplementary Material**. Further inquiries can be directed to the corresponding author.

## Ethics statement

The animal studies were approved by Institutional Animal Care and Use Committee of Japan BCG Laboratory. The studies were conducted in accordance with the local legislation and institutional requirements. Written informed consent was obtained from the owners for the participation of their animals in this study.

## Author contributions

SM: Conceptualization, Investigation, Writing – original draft, Writing – review & editing. YC: Investigation, Writing – review & editing. YS: Investigation, Writing – review & editing. SH: Investigation, Writing – review & editing. TB: Investigation, Writing – review & editing. EY: Investigation, Writing – review & editing. KM: Conceptualization, Funding acquisition, Project administration, Supervision, Writing – original draft, Writing – review & editing.
