# Supplementary material for: Culture filtrate proteins from BCG act as adjuvants for cytotoxic T lymphocyte induction
Source: Front Immunol. 2023 Oct 18;14:1271228. doi: 10.3389/fimmu.2023.1271228 (PMC10622798; doi:10.3389/fimmu.2023.1271228)
Supplement: Supplementary file 1 [file DataSheet_1.docx]

Supplementary Material

# Supplementary Figures


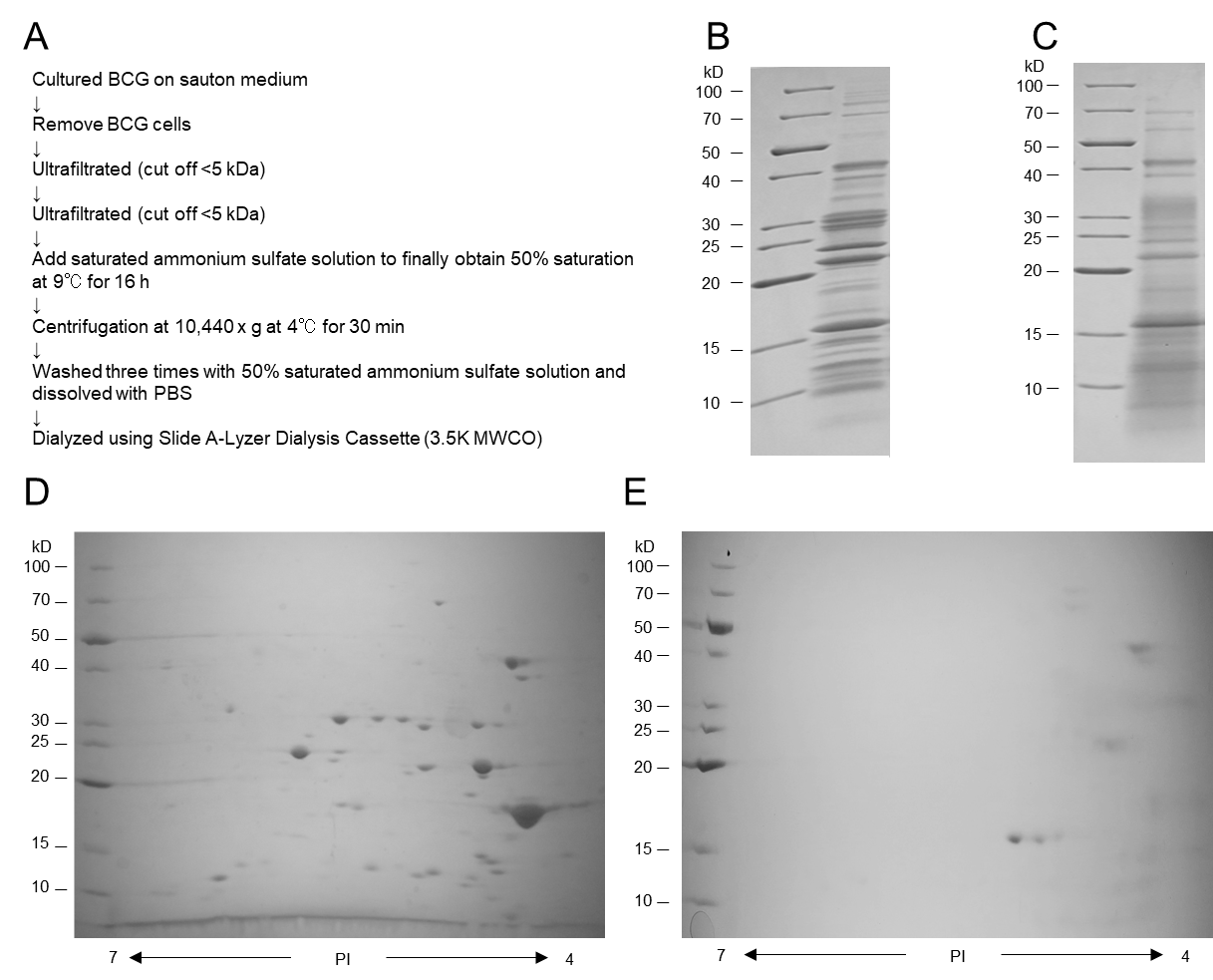


**Supplementary Figure 1.** Preparation of CFP. (A) Flow chart demonstrating CFP preparation. (B, C) SDS-PAGE analysis for CFP (B) or CFPH (C). (D, E) Two-dimensional PAGE analysis for CFP (D) or CFPH (E). Vertical and horizontal axes show fractionation by molecular mass and isoelectric point, respectively.

**
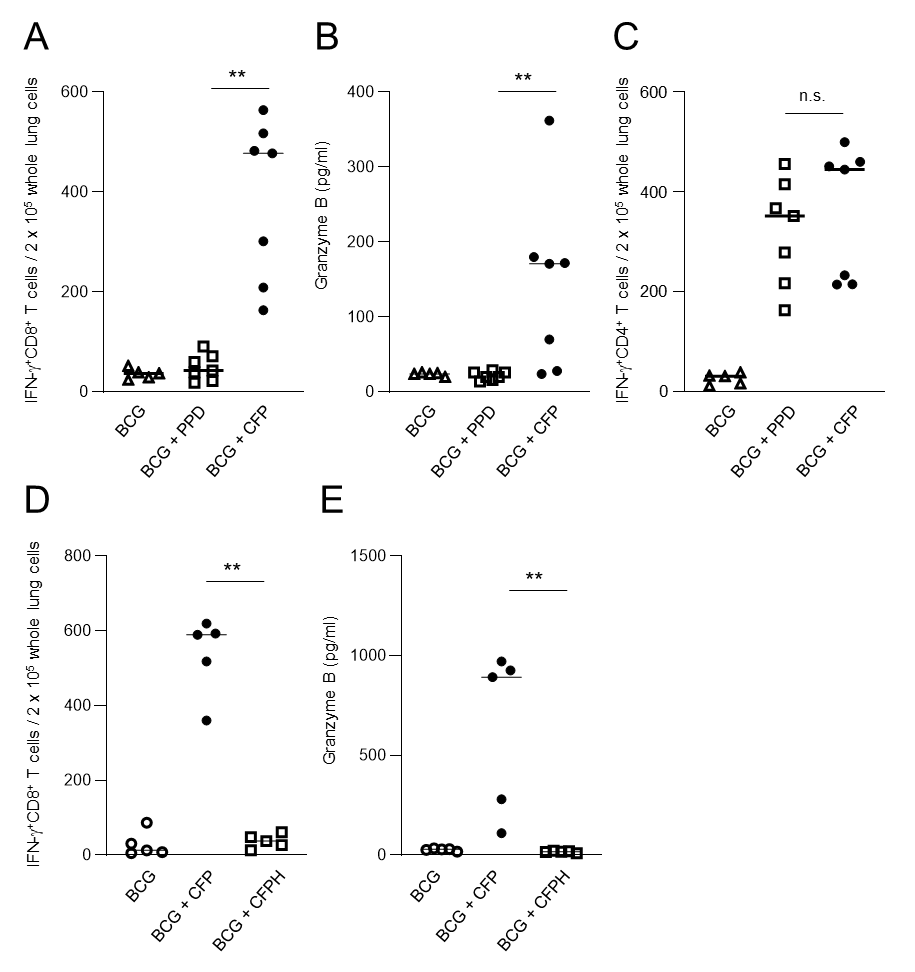
**

**Supplementary Figure 2**. Antigen-specific CTL can be induced only with CFP, not with PPD or CFPH. C57BL/6j mice were administrated with BCG-Tokyo (0.1 mg/mouse) by subcutaneous injection. Six weeks after BCG priming, the mice were boosted with 50 µg of CFP, PPD (A, B, C) or CFPH (D, E) three times with 2 weeks intervals. Two weeks after the last boost, lung was removed and used for the preparation of cell suspensions at concentrations of 2 x 10^5^ cells/well in the culture medium and stimulated with GM10 peptide (A, D) or peptide 25 (C) on ELISPOT plates. After incubation at 37℃ for 24 h, the plates were counted using KS ELISPOT. The granzyme B concentrations in the culture supernatant was measured by ELISA (B, E).

**
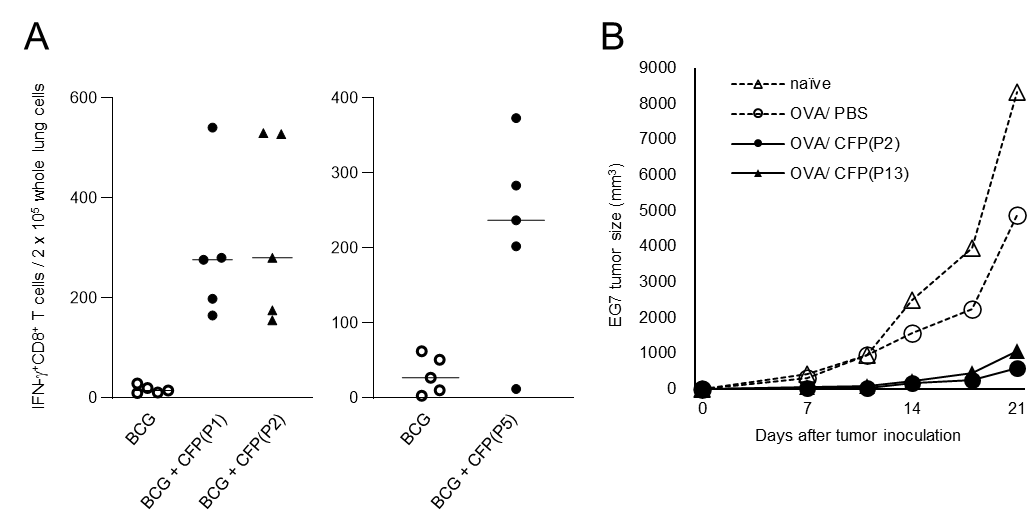
**

**Supplementary Figure 3.** CTL adjuvanticity of different lots of CFP. (A) Immunization of C57BL/6j mice, preparation of lung cell suspension, and measurement of spot forming cells were performed as described in the legend for supplementary Figure 2. P1, P2 and P5 indicated lot numbers of CFP. (B) C57BL/6j mice were immunized by subcutaneous injection on the abdomen with OVA (10 µg/mouse) with or without CFP for various lot (200 µg/mouse) at day 0 and day 10. Seven days after second immunization, mice were transplanted subcutaneously with EG7 (1 x 10^6^ cells/head) on their ventral. Tumor size measurement was described in the Methods section. P2 and P13 indicated lot numbers of CFP. Dotted lines with open triangle and with open circle indicate naïve mice and control group, respectively. Solid circles and Solid triangle indicate OVA/CFP(P2) and OVA/CFP(P13) group.
